# A Multidomain Longitudinal Analysis of Frailty, Functional Limitation, Balance and Falls in Older Adults

**DOI:** 10.3390/healthcare14132019

**Published:** 2026-07-07

**Authors:** Hammad S. Alhasan

**Affiliations:** Department of Medical Rehabilitation Sciences, Faculty of Applied Medical Sciences, Umm Al-Qura University, Makkah 24382, Saudi Arabia; hshasan@uqu.edu.sa

**Keywords:** falls, older adults, frailty, activities of daily living, balance, longitudinal study

## Abstract

**Background/Objectives**: Falls are a major threat to healthy ageing and the preservation of independence, yet the risk of falls in older adults commonly arises from multiple interacting domains rather than a single factor. This longitudinal analysis assessed whether frailty, functional limitation, and balance performance were associated with future falls among older adults. **Methods**: This longitudinal analysis assessed older adults aged 65 years and older over a three-year follow-up interval, using baseline data collected in 2015 and follow-up falls data collected in 2018. Baseline health, functional, and physical performance measures were evaluated in relation to self-reported falls at follow-up. Candidate predictors comprised sociodemographic, health, functional, and physical performance variables. Univariable logistic regression, adjusted screening models, and a final complete-case multivariable logistic regression model were used. **Results**: The eligible analytic sample comprised 1932 participants, of whom 480 (24.8%) reported falls at follow-up. In adjusted analyses, greater frailty index, greater ADL limitation, female sex, shorter full-tandem balance time, depressive symptoms, and greater self-rated mobility severity were associated with higher odds of future falls. However, only frailty index, ADL limitation, full-tandem balance time, and female sex were retained in the final multivariable model. In the final multivariable model of 1451 participants, frailty index per 0.1-unit increase (OR 1.26, 95% CI 1.07–1.48), ADL limitation score per 1-point increase (OR 1.30, 95% CI 1.16–1.45), female sex (OR 1.62, 95% CI 1.17–2.25) and longer full-tandem balance time per 10 s increase (OR 0.89, 95% CI 0.82–0.96) remained associated with falls. The final model showed modest discrimination and acceptable apparent calibration. **Conclusions**: Higher frailty, greater ADL limitation, poorer tandem balance and female sex were associated with subsequent falls. These results reinforce the value of a multidomain approach to fall-risk assessment.

## 1. Introduction

Population ageing is accelerating worldwide, and falls are now recognized as a major threat to healthy ageing and the maintenance of independent living [1,2]. Among older adults, approximately one quarter to one third experience at least one fall each year, with global estimates near 26.5% and some public health reports indicating annual fall proportions of approximately 28–35% [3,4]. The burden is even greater in advanced age, with approximately half of adults aged 80 years and older estimated to experience at least one fall annually [5]. Falls are a leading cause of unintentional injury-related mortality in older adults, with deaths often arising from fall-related injuries and subsequent complications or sequelae rather than from the fall incident itself [6,7,8]. These complications may include fractures, traumatic injury, immobility, hospitalization, functional decline, disability, institutionalization, loss of confidence, loss of independence, and increased mortality [8,9,10]. Accordingly, contemporary falls guidelines emphasize multidomain risk assessment rather than reliance on any single clinical characteristic [11].

Falls in later life rarely arise from a single isolated factor. Instead, they generally arise from the interplay of declining physiological reserve, functional limitation, impaired balance, chronic disease, decreased muscle strength, medication burden, cognitive impairment, neurological disease, sensory impairments, depressive symptoms, fear of falling, behavioural factors, and environmental hazards [12,13,14]. These domains may interact by reducing physiological reserve, impairing postural control, restricting safe mobility, or greater exposure to hazardous situations. Among these risk domains, frailty, functional limitation, and balance performance are especially relevant to clinical fall-risk evaluation because they reflect related but distinct dimensions of vulnerability: physiological reserve, daily functional capacity, and postural control [15,16]. Recent studies indicate that frailty is an important risk factor for falls, and that female sex, depression, and balance disorders also show consistent associations with increased fall risk in community-dwelling older adults [17,18,19,20]. Concurrently, objective balance assessment remains a core component of fall-risk evaluation, and recent longitudinal studies have further reinforced the predictive value of standing balance testing for future falls [21,22].

Foundational frailty research provides an important basis for this approach. Fried et al. defined frailty as a physical phenotype marked by weakness, slowness, exhaustion, low physical activity, and unintentional weight loss, and demonstrated that frailty was distinct from comorbidity and disability while predicting adverse outcomes in older adults [23]. In contrast, Rockwood and Mitnitski developed the deficit-accumulation model, in which frailty is expressed as the proportion of accumulated health deficits spanning symptoms, diseases, impairments, disabilities, and functional limitations [24]. These two landmark frameworks highlight that frailty may be understood both as a physical syndrome and as a multidimensional accumulation of vulnerability [25].

The present study used a deficit-accumulation frailty index, which is particularly appropriate for large ageing cohort datasets because it can synthesize multiple health deficits into a continuous measure of vulnerability [24,25]. However, frailty, activities of daily living (ADL) limitation, and balance performance should not be considered equivalent. Frailty captures broad physiological vulnerability, ADL limitation reflects the functional impact of vulnerability on essential daily activities, and tandem balance provides an objective performance-based assessment of postural control. Examining these domains simultaneously allows assessment of whether each contributes distinct information to subsequent falls after accounting for the others.

In particular, impairment in ADL and frailty are closely related in older adults, yet they are not interchangeable constructs. Recent longitudinal evidence suggests that frailty and ADL disability are bidirectionally related, whereas other studies suggest that ADL impairment may partly explain why vulnerable older adults are at greater risk of falling [26,27]. Establishing whether frailty and ADL limitation provide overlapping or independent information is therefore important for fall-risk assessment and prevention.

Although evidence from CHARLS has increased in recent years, several important gaps remain. Previous studies have frequently focused on single domains, such as depressive symptoms, pain, or frailty status, or generalized fall-risk prediction models, rather than investigating frailty, functional limitation, and specific balance performance jointly in the same longitudinal framework [26,28]. Recent studies using CHARLS data have shown that depressive symptoms are associated with subsequent falls, that musculoskeletal pain may contribute to increased future fall risk, and that frailty is positively related to falls [13,29]. However, these studies largely focused on individual exposures, mediating mechanisms, or prediction tools rather than identifying which clinically relevant domains remain independently associated with falls after mutual adjustment. Moreover, many analyses have been conducted in mixed samples of middle-aged and older adults, whereas fewer analyses have targeted adults aged 65 years and older, in whom fall burden is highest.

Emerging evidence also highlights the need for comprehensive and longitudinal analyses. For example, prior research has shown that standing balance tests may help predict future falls; however, these studies were undertaken in more specific community samples and did not evaluate broader frailty, ADL limitation, and tandem balance performance within the same analysis [13,30]. Therefore, longitudinal studies are needed to assess whether frailty, functional limitation, and balance offer distinct contributions to future falls in older adults.

To address these gaps, the present study used a secondary longitudinal analysis of CHARLS data to assess whether baseline frailty, ADL limitation, and tandem balance performance were associated with subsequent falls. The primary aim was to examine the mutually adjusted associations of these clinically relevant domains with future falls. As a secondary assessment, model discrimination and calibration were evaluated to describe the ability of the model to distinguish fallers from non-fallers. We hypothesized that frailty, ADL limitation, and impaired tandem balance would each be associated with subsequent falls.

## 2. Materials and Methods

### 2.1. Study Design, Data Source, and Ethical Considerations

This study was a retrospective secondary longitudinal analysis of de-identified data from CHARLS. CHARLS is a nationally representative survey of middle-aged and older adults residing in mainland China and forms part of the broader family of Health and Retirement Study-type ageing surveys [31].

Ethical approval and informed consent procedures were obtained for the original CHARLS data collection rather than separately for the present secondary analysis. Ethical approval for CHARLS was granted by the Institutional Review Board at Peking University. The approval number for the main household survey, including anthropometric assessments, was IRB00001052-11015, and the approval number for biomarker collection was IRB00001052-11014. During CHARLS fieldwork, respondents who agreed to participate signed informed-consent forms for the relevant survey components. Details of the CHARLS ethical approval and consent procedures are provided in the original CHARLS documentation and cohort profile.

CHARLS uses a stratified multistage probability-proportional-to-size sampling design, in which county-level units, village or urban community clusters, and households are sequentially selected. In each eligible household, one respondent aged 45 years or older is randomly selected, and his or her spouse is also included. CHARLS data were obtained through face-to-face computer-assisted personal interviews, as described in the original CHARLS cohort profile [31].

For the present analysis, baseline measures collected in 2015 were evaluated in relation to falls reported at the 2018 follow-up, representing an approximately three-year follow-up interval. A respondent-level wide-format dataset was constructed using the unique participant identifier to link baseline variables with follow-up falls status. Records were examined to confirm that each participant contributed only one observation.

### 2.2. Study Population and Eligibility Criteria

The analytic cohort was restricted to participants aged 65 years or older at baseline who had a valid participant identifier and non-missing follow-up falls status. Participants were excluded if they were younger than 65 years at baseline or had missing follow-up falls information. Missing covariate data were handled as described in the Section 2.4.

The age threshold of 65 years was selected because the study aim was to examine fall-related risk domains among participants who were already within the conventional older-adult age range at the time frailty, functional limitation, and balance performance were measured. Although CHARLS includes adults younger than 65 years and some public-health definitions use lower thresholds for older adulthood, this study focused on an established older-adult cohort rather than adults transitioning from late middle age into older age. The ≥65-year threshold also improves comparability with much of the gerontological and fall-risk literature, where adults aged 65 years and older are commonly considered a high-risk population for falls.

### 2.3. Measures

#### 2.3.1. Subsequent Falls

The primary outcome was self-reported falls at the 2018 follow-up assessment. Participants were classified as having subsequent falls if they reported one or more falls during the period since the previous interview.

#### 2.3.2. Frailty Index

Frailty index was used as a continuous deficit-accumulation measure, with higher scores reflecting greater frailty. For the 2015 baseline assessment, the index included up to 24 deficits covering 14 chronic diseases, three mobility items, ADL limitation, IADL limitation, depressive symptoms, sleep-related symptoms, orientation, figure drawing, and physical activity. Because the frailty index may conceptually overlap with measures of functional limitation, collinearity diagnostics and sensitivity analyses were undertaken to examine the stability of findings involving frailty and ADL limitation. A prespecified set of baseline deficits covering chronic disease, mobility, ADL/IADL limitation, depressive symptoms, sleep-related symptoms, cognition, and low physical activity was defined, and the index was calculated by dividing the sum of available deficit scores by the number of non-missing deficit items, yielding a continuous score from 0 to 1 with higher values indicating greater frailty [25,32]. Frailty index was rescaled per 0.1-unit increase. The full list of deficits included in the wave 3 frailty index is provided in Table A1.

#### 2.3.3. Functional Limitation and Balance Performance

ADL limitation score was calculated by summing difficulty across six ADLs: dressing, bathing, eating, getting in and out of bed, toileting, and continence. Scores ranged from 0 to 6, with higher scores reflecting greater limitation. Standing balance performance measures included side-by-side, semi-tandem, and full-tandem balance tests. Full-tandem balance time was selected for the final model as a performance-based measure of postural control. In regression analyses, full-tandem balance time was rescaled per 10 s increase to enhance interpretability of odds ratios.

#### 2.3.4. Covariates and Variable Preparation

The study considered the following sociodemographic covariates: age, sex, educational attainment, marital status, institutional living status, and current work status. Lifestyle variables included current smoking and alcohol use in the last year. Age was analysed as a continuous variable, and sex was analysed as a binary categorical variable. Marital status was regrouped into coupled, widowed, and other uncoupled categories before final modelling to minimize sparse-cell instability and improve interpretability.

Other candidate predictors were extracted from directly available CHARLS variables, including self-rated mobility severity, depressive symptoms score (CESD-10), systolic blood pressure, waist circumference, body mass index (BMI), grip strength, walking speed time, five-chair-stands time, and standing balance performance measures (side-by-side, semi-tandem, and full-tandem balance tests). Medication burden was defined as the baseline number of medication-use indicators available in CHARLS for chronic disease management and was modelled as a continuous covariate in the final multivariable model, with higher values indicating a greater number of medication-use indicators.

Variables were coded and standardized before analysis using prespecified definitions. Smoking was treated as a categorical variable. As implausible extreme BMI values were detected during preliminary screening, a cleaned BMI variable was derived for inferential analyses by coding implausible values outside 10 to 60 kg/m^2^ as missing. In this study, implausible BMI values were defined as values outside a prespecified adult plausibility range that were unlikely to represent valid BMI measurements in a community-based older-adult survey and were more likely to reflect data-entry, measurement, or unit-conversion errors [33]. The lower bound of 10 kg/m^2^ was selected because it is far below established adult severe-thinness thresholds; WHO identifies BMI < 16 kg/m^2^ as an extreme limit associated with markedly increased health risk. The upper bound of 60 kg/m^2^ was selected because it is substantially above the conventional threshold for class III obesity and has been used as an upper plausibility threshold in epidemiological BMI data cleaning. Values outside this range were therefore treated as missing rather than retained in the cleaned BMI variable.

### 2.4. Statistical Analysis

Statistical analyses were conducted using IBM SPSS Statistics version 29.0 and R version 4.4.2. Baseline characteristics were summarized using means and standard deviations, medians and interquartile ranges, or frequencies and percentages, as appropriate. Between-group comparisons according to follow-up falls status were performed using chi-square tests, independent-samples *t* tests, or Mann–Whitney U tests, depending on variable type and distribution.

Logistic regression was used to examine associations between baseline predictors and subsequent falls. Although CHARLS uses a stratified multistage sampling design, the present analyses did not incorporate survey weights, clustering, or stratification. Therefore, the regression models were interpreted as analytic-sample association models rather than design-based population estimates. Because the exact timing of the first fall during follow-up was unavailable, time-to-event analysis and incidence-rate modelling were not feasible; therefore, falls were analysed as a binary follow-up outcome using logistic regression, and associations were reported as odds ratios.

Univariable models were first fitted for each candidate predictor, followed by adjusted screening models controlling for age, sex, current smoking, and cleaned BMI.

A final complete-case multivariable logistic regression model was then fitted using clinically relevant and consistently associated predictors. The final complete-case model included frailty index, ADL limitation score, full-tandem balance time, age, sex, smoking status, BMI, alcohol use, education, marital status, and medication burden. Self-rated mobility severity was not retained because it conceptually overlapped with ADL limitation and full-tandem balance time. CESD-10 was not included in the final model because of higher missingness and the resulting reduction in complete-case sample size; therefore, it was assessed in a sensitivity analysis. Exploratory sex-specific analyses were conducted as supplementary analyses. The univariable logistic regression models, adjusted screening models, and final multivariable logistic regression model were repeated separately among men and women. In these stratified models, sex was not included as a covariate. The adjusted screening models controlled for age, current smoking, and BMI, except when the predictor being tested was one of these adjustment variables.

Model discrimination was assessed using the area under the receiver operating characteristic curve (ROC), and calibration was assessed using the Brier score, calibration intercept, calibration slope, calibration plot, and Hosmer–Lemeshow test. Collinearity was examined using correlation diagnostics and variance inflation factors.

The primary final model used complete-case analysis. Missingness was summarized for all model variables, and participants included in the complete-case model were compared with those excluded because of missing covariate data. Multiple imputation by chained equations using 20 imputed datasets was conducted as a sensitivity analysis for missing covariate data. The imputation model comprised the outcome, all variables in the final multivariable model, and IADL limitation score as an auxiliary variable. Logistic regression estimates from imputed datasets were synthesized using Rubin’s rules.

Additional sensitivity analyses were conducted to assess the potential overlap between frailty and ADL limitation. These included models omitting ADL limitation or frailty index, and a modified frailty-index model in which the ADL component was removed from the frailty-index numerator and denominator before recalculating the index. CESD-10 score was evaluated in the unadjusted and adjusted screening analyses but was not included in the primary final complete-case model because of higher missingness and the resulting reduction in complete-case sample size. CESD-10 had 392 missing observations among the 1932 eligible participants (20.3%). Including CESD-10 in the final complete-case model would have reduced the analytic sample from 1451 to 1202 participants. Therefore, CESD-10 was evaluated in an additional sensitivity analysis rather than included in the primary final model.

All statistical tests were two-sided, and *p* < 0.05 was considered statistically significant. Additional details on variable preparation, distributional assessment, collinearity diagnostics, missing-data handling, and sensitivity analyses are provided in Appendix A.

## 3. Results

### 3.1. Participant Characteristics

**Figure 1:**
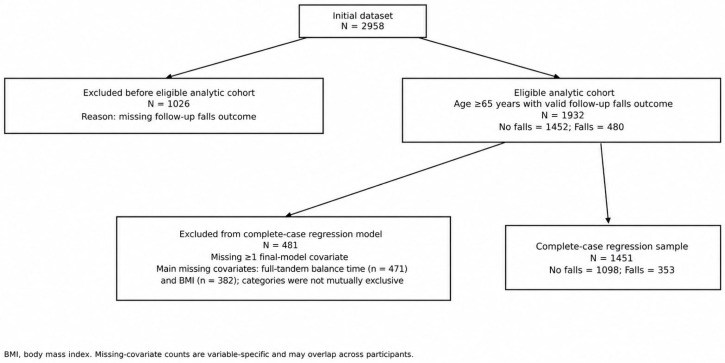
Participant selection flow diagram.

The study comprised 1932 participants aged 65 years and older with complete follow-up information on falls. Overall, 480 participants (24.8%) reported falls and 1452 (75.2%) did not. The participant selection process is shown in Figure 1. Mean age was slightly higher among fallers than non-fallers (72.5 ± 6.4 vs. 72.0 ± 6.1 years; *p* = 0.118), and systolic blood pressure was also slightly higher (132.6 ± 20.7 vs. 130.5 ± 21.1 mmHg; *p* = 0.086), although neither comparison was statistically significant.

In contrast, frailty index (0.30 ± 0.16 vs. 0.23 ± 0.16; *p* < 0.001), ADL limitation score (1.14 ± 1.61 vs. 0.48 ± 1.10; *p* < 0.001), and IADL limitation score (1.11 ± 1.47 vs. 0.67 ± 1.18; *p* < 0.001) were all higher among fallers.

Female participants accounted for 62.9% of fallers compared with 47.4% of non-fallers (*p* < 0.001). Marital status differed significantly between fallers and non-fallers, with fallers being less likely to be married and more likely to be widowed (*p* = 0.019). Fallers were less likely to be current smokers than non-fallers (17.3% vs. 28.4%; *p* < 0.001), and sarcopenia was more prevalent among fallers (41.5% vs. 34.4%; *p* = 0.014). No significant between-group differences were observed for education, living status, current work, and alcohol use in the last year. For detailed baseline characteristics, refer to Table 1.

### 3.2. Missing-Data Assessment

Participants excluded from the complete-case analysis showed a marginally higher fall proportion than included participants, though this difference was not statistically significant (26.4% vs. 24.3%, *p* = 0.394). Excluded participants were slightly older, were more often women, and had greater frailty, ADL limitation, and IADL limitation scores. The final complete-case model comprised 1451 participants, while 481 participants from the eligible analytic cohort were excluded because of missing data in one or more model covariates. The comparison between participants included in and excluded from the complete-case model is presented in Table A2. Therefore, multiple imputation was conducted as a sensitivity analysis to assess whether missing covariate data influenced the main findings.

### 3.3. Collinearity Diagnostics

Collinearity diagnostics did not show problematic multicollinearity. Adjusted GVIF values were below 2 for all predictors (Table A3). The correlation between frailty index and ADL limitation score was moderate (r = 0.444) and the highest observed correlation was between frailty index and medication burden (r = 0.677). These findings suggest related but not statistically redundant constructs (Table A4).

### 3.4. Univariable and Adjusted Screening Logistic Regression Analyses

In unadjusted analyses, several demographics, functional, frailty-related, and physical performance variables were linked to subsequent falls. Female sex, widowhood, sarcopenia, greater frailty index, greater ADL limitation, and higher depressive symptoms were associated with increased odds of falls, whereas higher grip strength and longer full-tandem balance duration were associated with reduced odds. Detailed unadjusted estimates are presented in Table 2.

After adjustment for age, sex, current smoking, and BMI, the most consistent relationships were identified for frailty index, ADL limitation score, female sex, full-tandem balance duration, self-rated mobility difficulty, and depressive symptoms. Some associations identified in unadjusted analyses, including widowhood, grip strength, and sarcopenia, were attenuated after adjustment. Detailed adjusted estimates are presented in Table 2.

### 3.5. Final Multivariable Model

The final complete-case multivariable model included 1451 participants, of whom 353 reported subsequent falls. Each 0.1-unit increase in frailty index was associated with greater odds of falls (OR 1.26, 95% CI 1.07–1.48, *p* = 0.004). Each 1-point increase in ADL limitation score was also associated with greater odds of falls (OR 1.30, 95% CI 1.16–1.45, *p* < 0.001). Longer full-tandem balance time was associated with lower odds of falls (OR 0.89 per 10 s increase, 95% CI 0.82–0.96, *p* = 0.005). Women had higher odds of falls than men (OR 1.62, 95% CI 1.17–2.25, *p* = 0.004). Age, BMI, smoking status, alcohol use, education, marital status, and medication burden were not associated with falls in this model.

**Figure 2:**
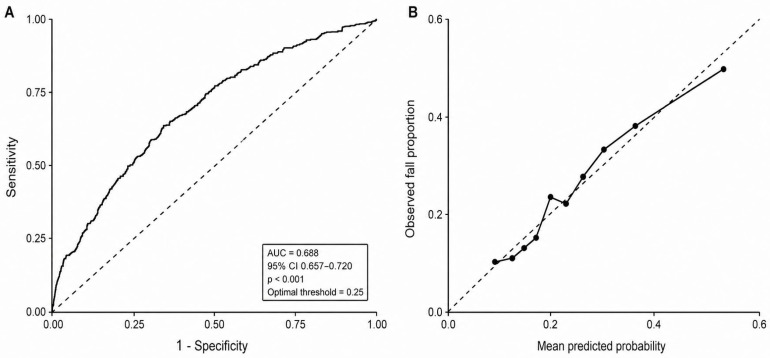
Discrimination and calibration of the final complete-case model. (**A**) Receiver operating characteristic curve for the final complete-case multivariable model. The dashed diagonal line indicates no discrimination. (**B**) Calibration plot for the final complete-case model. Predicted probabilities were grouped into deciles. Points represent observed fall proportions within deciles, and the dashed diagonal line represents perfect calibration.

The final complete-case model showed modest discrimination, with an AUC of 0.688 (95% CI 0.657–0.720). The Brier score was 0.168, and apparent calibration was acceptable, with calibration intercept 0.000, calibration slope 1.000, and Hosmer–Lemeshow *p* = 0.606. The ROC and calibration plot are presented in Figure 2. The full final multivariable model is shown in Table 3.

### 3.6. Sex-Specific Analyses

Sex-specific analyses were conducted within the primary complete-case analytic sample and are presented in Table A5, Table A6 and Table A7. The analytic sample included 730 men, of whom 126 reported subsequent falls, and 721 women, of whom 227 reported subsequent falls. ADL limitation, frailty index, and IADL limitation were associated with subsequent falls in both men and women. Full-tandem balance time and medication burden were more clearly associated with falls among women.

In the sex-specific final multivariable models, frailty index and ADL limitation remained significantly associated with subsequent falls among men, whereas full-tandem balance showed a borderline association. Among women, ADL limitation and full-tandem balance remained significantly associated with subsequent falls, whereas the association for frailty index was attenuated and did not reach conventional statistical significance.

### 3.7. Sensitivity Analyses

Sensitivity analyses were conducted to assess the potential overlap between frailty and ADL limitation. When ADL limitation was omitted from the model, frailty index remained associated with subsequent falls. When frailty index was excluded from the model, ADL limitation remained associated with subsequent falls. Longer full-tandem balance time and female sex also remained associated with later falls across sensitivity models. Full frailty/ADL overlap sensitivity results are shown in Table 4. Multiple-imputation analysis using 20 imputed datasets showed results consistent with the complete-case model. Full multiple-imputation model results are shown in Table 5.

An additional sensitivity analysis was performed to examine direct measurement overlap between the frailty index and ADL limitation. In this analysis, the frailty index was recalculated after excluding the ADL component, and the final multivariable model was refitted while retaining ADL limitation score as a separate predictor. ADL limitation remained strongly associated with subsequent falls (OR = 1.36, 95% CI 1.23–1.51, *p* < 0.001). Longer full-tandem balance time remained associated with lower odds of falls (OR = 0.89, 95% CI 0.82–0.97, *p* = 0.008), and women had higher odds of falls than men (OR = 1.69, 95% CI 1.22–2.34, *p* = 0.002). The modified frailty index excluding ADL was attenuated and did not reach conventional statistical significance (OR = 1.11, 95% CI 1.00–1.24, *p* = 0.056). Full results are shown in Table 6.

Another sensitivity analysis was conducted by adding CESD-10 score to the final multivariable model. In this model, CESD-10 was not statistically significant after adjustment for the final-model covariates (OR 1.02, 95% CI 1.00–1.04, *p* = 0.080). Frailty index, ADL limitation score, full-tandem balance time, and female sex remained associated with subsequent falls. Full results are shown in Table 7.

## 4. Discussion

In this longitudinal analysis of older adults aged 65 years and older, higher frailty index, greater ADL limitation, shorter full-tandem balance time, and female sex were independently associated with future falls. These associations were maintained after adjustment for available sociodemographic, behavioural, anthropometric and clinical covariates and were generally supported by sensitivity analyses. The final model exhibited acceptable apparent calibration but modest discrimination, suggesting that these variables are useful for explaining longitudinal fall risk but do not provide sufficient predictive accuracy on their own.

These findings indicate that future falls in older adults are more strongly related to multidimensional vulnerability and functional impairment than to demographic factors alone. Notably, frailty, ADL limitation, and tandem balance may each capture distinct but complementary aspects of fall susceptibility. Frailty may reflect broader physiological vulnerability, ADL limitation may reflect the functional impact of reduced reserve, and tandem balance provides a simple objective indicator of postural control. The retention of all three variables in the final model suggests that they capture related but distinct constructs.

### 4.1. Comparison with Previous Literature

The observed association between frailty and future falls in the current study is consistent with recent evidence indicating that frailty is a major geriatric syndrome relevant to fall risk. A 2023 meta-analysis identified frailty as a significant risk factor for falls in older adults, although the strength of the association varies across frailty instruments [34]. This association is clinically plausible because the frailty index captures cumulative physiological vulnerability across multiple health domains rather than a single impairment [35]. Older adults with higher frailty may have slower protective reactions, poorer mobility confidence, greater comorbidity burden, and limited ability to compensate when exposed to balance challenges.

The association observed for ADL limitation is also in line with recent longitudinal evidence demonstrating temporal links between disability and frailty. Previous studies have demonstrated a bidirectional relationship between frailty and ADL or IADL disability in middle-aged and older adult populations, suggesting that these constructs evolve together but are not identical [36]. ADL limitation may reflect the practical expression of vulnerability during daily life. Difficulty with dressing, bathing, toileting, transferring, eating, or continence may suggest reduced strength, coordination, endurance, mobility, and environmental adaptation [37]. ADL limitation may also increase fall risk because these routine activities involve transfers, turning, reaching, stepping, and postural-control requirements. Our results contribute to this literature by showing that ADL limitation remained independently associated with future falls after frailty was included in the same model. This interpretation suggests that functional difficulty in daily activities may be a relevant pathway through which broader vulnerability manifests as increased fall risk. Therefore, ADL limitation may offer clinically useful information beyond the frailty index because it captures how physiological vulnerability influences everyday movement and independence.

The sensitivity analysis employing a modified frailty index with the ADL component excluded provides a more conservative evaluation of overlap between frailty and functional limitation. This analysis indicates that part of the association identified for the original frailty index may have been partly attributable to direct overlap with functional limitation. Therefore, the findings should be viewed as showing that ADL limitation, tandem balance, and female sex were the most consistent independent correlates of subsequent falls in the final model, while the independent effect of frailty beyond ADL limitation was less clear when direct ADL overlap was excluded.

The observed inverse association of longer full-tandem balance time aligns with recent literature. A longitudinal study found that standing balance testing can help detect older adults at increased risk of falls [38]. Concurrently, broader evidence suggests that single mobility or balance measures rarely demonstrate strong predictive performance [39]. Unlike self-reported function, tandem balance provides a direct observation of physical performance. Full-tandem stance represents an objective performance-based assessment that tests postural control by narrowing the base of support [38]. Maintaining this position depends on integration of sensory inputs, neuromuscular control, lower-limb stability, trunk control, and timely balance corrections. This interpretation is consistent with our findings, as tandem balance remained an independent correlate of falls, yet overall discriminative performance of the model remained modest. From a clinical perspective, these findings support the use of tandem balance as part of multidomain fall-risk assessment rather than as an isolated screening tool. Its primary value is likely as a simple, practical, objective clinical measure that complements frailty and ADL assessment within a multidomain fall-risk evaluation approach.

Female sex remained an independent predictor of future falls after adjustment, which is in line with prior falls literature reporting higher fall burden in women [9]. This finding is aligned with recent CHARLS-based evidence showing a higher fall burden among older women [40]. However, the current study adds to previous CHARLS analyses by focusing specifically on adults aged 65 years and older and by demonstrating that the association persisted after concurrent adjustment for frailty, ADL limitation, tandem balance performance, BMI, medication burden, and sociodemographic factors. Therefore, the higher odds of falls among women were not fully accounted for by the measured frailty, functional limitation, or balance domains included in this model. In the Chinese older-adult population, this residual association may indicate several unmeasured or partially captured factors, including sex differences in muscle strength and muscle quality, osteoporosis and fracture vulnerability, pain burden, footwear and household mobility exposures, caregiving and domestic activity patterns, and differences in fall reporting. National injury surveillance data from China also suggest that many falls among older adults occur at home and during leisure activities or household tasks, suggesting that everyday environmental and activity patterns may be important when interpreting sex differences in fall risk [41]. Accordingly, our findings should be interpreted as evidence that female sex identifies a subgroup with higher observed fall risk in this cohort, rather than as evidence that biological sex alone explains the association.

The sex-specific analyses offered additional context for the primary findings. ADL limitation was linked with subsequent falls in both men and women, reinforcing its role as a consistent functional marker of fall risk. Frailty index remained significant among men but was attenuated among women, whereas full-tandem balance was more clearly linked to falls among women. These findings indicate that the relative contribution of frailty and balance performance may vary by sex. However, these subgroup findings should be interpreted with caution because the sex-specific models had smaller sex-stratified samples and some categorical estimates were affected by sparse-data limitations.

Depressive symptoms were associated with later falls, which is in line with a previous longitudinal study showing that depression was associated with increased fall risk [26]. This finding is clinically plausible, as depressive symptoms may be linked to reduced physical activity, impaired concentration and poor balance confidence, all of which may increase susceptibility to falls [36,42,43]. In the current study, depressive symptoms remained associated with future falls in both unadjusted and adjusted regression analyses, indicating that mood-related vulnerability may constitute an important component of multidimensional fall risk. However, in the CESD-10 sensitivity analysis, CESD-10 was not statistically significant after adjustment for the final-model covariates, suggesting that its independent role within the final multidomain model was not confirmed.

Age was negatively correlated with full-tandem balance duration, indicating that older participants generally exhibited poorer tandem balance performance. Therefore, full-tandem balance time may have reflected part of the age-related variation in postural control, mobility reserve, and neuromuscular function that is relevant to falls. In this context, the non-significant age coefficient does not indicate that chronological age is unrelated to fall risk. Rather, after adjustment for frailty, ADL limitation, and a performance-based measure of balance, the remaining direct association between age and subsequent falls was weakened. This finding reinforces the clinical value of assessing functional and balance performance directly, rather than relying on chronological age alone.

### 4.2. Clinical and Public Health Implications

These findings have several practical implications. First, they support a multidomain approach to fall-risk assessment in older adults rather than approaches based only on age or demographic characteristics. In the current study, age did not retain an independent association with falls once frailty, ADL limitation, and balance were considered, indicating that biological and functional vulnerability may better capture fall risk than chronological age in this context.

The modest discrimination observed in the present study is consistent with the broader fall-prediction evidence base. A recent systematic review of fall prediction models for older adults reported that discriminative performance varied substantially across studies, with cohort-based models generally showing AUC values in the low-to-moderate range [44]. In this context, the AUC of 0.688 reported in the current study suggests that frailty, ADL limitation, tandem balance, and sex help characterize fall susceptibility but are not sufficient for precise individual-level prediction. This is clinically reasonable because falls are multifactorial events influenced not only by intrinsic vulnerability but also by medication burden, sensory impairment, environmental hazards, prior fall history, and acute health changes. Therefore, the model should be interpreted as identifying clinically meaningful domains associated with future falls rather than as a standalone prediction instrument.

From a clinical standpoint, the findings are most useful as markers of multidomain vulnerability rather than as isolated statistical predictors. A 0.1-unit increase in frailty index, a 1-point increase in ADL limitation score, and a 10 s reduction in full-tandem balance time are clinically interpretable markers that reflect broader vulnerability, reduced daily functional capacity, and impaired postural control. These domains are directly relevant to fall-risk assessment because they can be recognized in routine clinical or community settings. For clinicians, the results indicate that older adults with greater ADL limitation and poorer tandem balance should be prioritized for more comprehensive fall-risk assessment, functional rehabilitation, environmental review, and education regarding safe mobility. The association with female sex also indicates that older women in this cohort may require particular consideration during fall-risk screening, although sex should be interpreted as a risk marker rather than a modifiable cause.

Fall risk is shaped by contextual factors such as dietary patterns, healthcare availability, health-seeking behaviours, physical activity patterns, family and caregiving arrangements, environmental exposures, and cultural attitudes toward ageing and mobility [45,46,47,48]. Population variation in body composition, sarcopenia risk, chronic disease patterns, and rehabilitation access may also alter the relationships among frailty, ADL limitation, balance performance, and falls. Therefore, the findings should be interpreted primarily in relation to older adults represented within the CHARLS analytic cohort, and external validation in other populations is needed before broader application.

### 4.3. Strengths and Limitations

A key strength of this study is its longitudinal design in a large ageing cohort, which enabled baseline factors to be assessed in relation to subsequent falls. Another important strength lies in the study’s analytic strategy. Instead of focusing on a single exposure, the study considered a broad set of demographic, behavioural, frailty-related, functional, and performance variables, followed by the use of adjusted screening models, a final multivariable model, model-performance assessment, and sensitivity analysis to assess the robustness of the identified associations.

Several limitations should be acknowledged. First, although the final model showed acceptable apparent calibration, its discriminative performance was modest, limiting its usefulness as a stand-alone prediction model. Second, the final regression analyses were performed using a complete-case approach and the exclusion of participants with missing data may have introduced selection bias. Although multiple imputation produced similar results, the complete-case model remained the primary analysis and may still be influenced by selection bias if missingness was not random. Third, depressive symptoms were not included in the primary final model because CESD-10 had additional missingness. Although CESD-10 was evaluated in a sensitivity analysis and was not statistically significant after adjustment for the final-model covariates, the additional missingness in CESD-10 data still limits interpretation of the independent contribution of depressive symptoms.

Fourth, survey weights, clustering, and stratification were not accounted for in the regression analyses. As a result, the findings should not be interpreted as nationally representative weighted estimates for the broader population of older adults in China. The use of unweighted models may also influence standard-error estimation and limits the generalizability of the findings beyond the analytic sample. Fifth, the analysis was based solely on CHARLS. Although CHARLS provides a valuable large-scale longitudinal data source, the observed associations may not be directly generalizable to older adults in other countries or healthcare contexts.

Sixth, falls were identified by self-report at follow-up over an approximately three-year recall period. This may have introduced recall bias and outcome misclassification, particularly because minor or non-injurious falls are more likely to be forgotten or underreported over long recall periods. Such underreporting would lower the observed fall frequency. If underreporting was largely non-differential with respect to baseline frailty, ADL limitation and other predictors, the resulting misclassification would be expected to weaken the observed associations toward the null [49]. Therefore, the reported associations may underestimate the true relationships between baseline multidomain vulnerability and subsequent falls. However, if recall accuracy varied systematically by health, cognitive, or functional status, the direction and magnitude of bias may be less predictable.

Seventh, adults aged ≤64 years at baseline were not included, although some may have transitioned into the ≥65-year age group during follow-up. Therefore, the findings should not be generalized to early older adults or to individuals transitioning from late midlife into older age. Future analyses should investigate whether the associations differ when adults aged 60 years and older are included or when age-transition subgroups are analyzed separately.

Finally, several functional constructs were derived variables rather than single directly observed measures and their interpretation depends on the validity of the underlying operational definitions.

### 4.4. Implications for Future Research

Future research should assess whether these findings can be strengthened by integrating additional domains, such as medication burden, sensory impairment, pain and environmental exposures. Repeated-measures and trajectory-based analyses may help clarify how frailty, disability, depressive symptoms, and balance change over time before the onset of falls. From a clinical perspective, future studies should examine whether combining frailty screening, functional assessment, and brief balance testing can enhance early identification of high-risk older adults in community and primary care settings. Future studies should examine whether musculoskeletal health, sarcopenia severity, pain distribution, home environment, and activity exposure explain or modify the sex difference.

## 5. Conclusions

In this secondary longitudinal analysis of older adults, frailty, ADL limitation, tandem balance performance, and female sex were associated with subsequent falls. These findings support a multidomain approach to fall-risk assessment and highlight the importance of considering physiological vulnerability, functional limitation, and balance performance together. However, the findings should be interpreted as longitudinal associations and not as evidence of a validated high-accuracy prediction model.

## Figures and Tables

**Table 1:** Baseline demographic, social, behavioral, and clinical characteristics of participants.

Characteristic	No Falls (n = 1452)	Falls (n = 480)	*p* Value
**Sex, n (%)**			**<0.001**
Men	764 (52.6)	178 (37.1)	
Women	688 (47.4)	302 (62.9)	
**Education level, n (%)**			0.136
Less than lower secondary education	1376 (94.8)	463 (96.5)	
Upper secondary education and vocational training	58 (4.0)	10 (2.1)	
Tertiary education	18 (1.2)	7 (1.5)	
**Living in nursing home or hospital at time of interview, n (%)**			0.778
No	1438 (99.0)	477 (99.4)	
Yes	14 (1.0)	3 (0.6)	
**Sarcopenia status, n (%)**			**0.014**
No sarcopenia	873 (60.1)	262 (54.6)	
Sarcopenia	499 (34.4)	199 (41.5)	
Missing	80 (5.5)	19 (4.0)	
**Marital status, n (%)**			**0.019**
Married	1029 (70.9)	310 (64.6)	
Partnered	41 (2.8)	8 (1.7)	
Separated	3 (0.2)	0 (0.0)	
Divorced	15 (1.0)	5 (1.0)	
Widowed	351 (24.2)	149 (31.0)	
Never married	13 (0.9)	8 (1.7)	
**Currently working, n (%)**			0.276
No	700 (48.3)	245 (51.1)	
Yes	750 (51.7)	234 (48.9)	
**Current smoking, n (%)**			**<0.001**
No current smoking	1039 (71.6)	396 (82.7)	
Current smoking	413 (28.4)	83 (17.3)	
**Alcohol use in the last year, n (%)**			0.083
No	997 (68.7)	349 (72.9)	
Yes	455 (31.3)	130 (27.1)	
Age, years, mean ± SD	71.96 ± 6.08	72.46 ± 6.35	0.118
Systolic BP, mmHg, mean ± SD	130.45 ± 21.07	132.55 ± 20.71	0.086
Frailty index, median (IQR)	0.232 (0.166)	0.301 (0.169)	**<0.001**
ADL limitation score, mean ± SD	0.48 ± 1.10	1.14 ± 1.61	**<0.001**
IADL limitation score, mean ± SD	0.67 ± 1.18	1.11 ± 1.47	**<0.001**

Abbreviations: SD, standard deviation; BP, blood pressure; IQR, interquartile range; ADL, activities of daily living. Continuous variables are presented as mean ± SD or median (IQR), as appropriate. Categorical variables are presented as n (%). *p* values of significant differences are presented in bold (*p* < 0.05). Percentages are calculated using non-missing denominators unless a missing category is explicitly shown.

**Table 2:** Univariable and adjusted screening logistic regression analyses of baseline predictors of falls among participants.

Predictor	Comparison/Unit	Unadjusted Models		Adjusted Models	
		OR (95% CI)	*p* Value	OR (95% CI)	*p* Value
Sex	Women vs. men	1.88 (1.52–2.33)	**<0.001**	1.81 (1.37–2.38)	**<0.001**
Age (years)	Per 1-year increase	1.01 (0.99–1.03)	0.118	1.02 (0.99–1.04)	0.134
Marital status	Widowed vs. married	1.41 (1.12–1.77)	**0.003**	1.12 (0.85–1.49)	0.426
Currently working	Yes vs. no	0.89 (0.73–1.10)	0.276	1.11 (0.87–1.42)	0.398
Current smoking	Yes vs. no	0.53 (0.41–0.69)	**<0.001**	0.70 (0.50–0.99)	**0.044**
Alcohol use in last year	Yes vs. no	0.82 (0.65–1.03)	0.083	1.16 (0.88–1.52)	0.293
Sarcopenia	Yes vs. no	1.33 (1.07–1.65)	**0.009**	1.25 (0.98–1.59)	0.071
Systolic blood pressure	Per 1 mmHg increase	1.01 (0.99–1.01)	0.086	1.00 (0.99–1.01)	0.282
Waist circumference	Per 1 cm increase	0.99 (0.99–1.00)	0.362	0.99 (0.99–1.00)	0.251
Frailty index	Per 0.1-unit increase	1.41 (1.30–1.53)	**<0.001**	1.43 (1.30–1.57)	**<0.001**
ADL limitation score	Per 1-point increase	1.42 (1.32–1.53)	**<0.001**	1.42 (1.31–1.55)	**<0.001**
Grip strength	Per 1 kg increase	0.97 (0.96–0.99)	**<0.001**	0.99 (0.98–1.01)	0.207
Walking speed time	Per 1 s increase	1.05 (1.00–1.11)	0.063	1.02 (0.96–1.08)	0.572
5-chair-stands time	Per 1 s increase	1.02 (0.99–1.06)	0.142	1.00 (0.97–1.04)	0.865
Side-by-side stand time	Per 1 s increase	0.80 (0.47–1.35)	0.397	0.88 (0.46–1.69)	0.697
Semi-tandem balance time	Per 1 s increase	0.80 (0.62–1.04)	0.092	0.82 (0.64–1.06)	0.129
Full-tandem balance time	Per 1 s increase	0.99 (0.98–0.99)	**<0.001**	0.99 (0.98–0.99)	**0.001**
Self-rated mobility severity score	Per 1-point increase	1.04 (0.90–1.22)	0.589	1.20 (1.11–1.30)	**<0.001**
Depressive symptoms score (CESD-10)	Per 1-point increase	1.05 (1.03–1.07)	**<0.001**	1.05 (1.03–1.07)	**<0.001**

Abbreviations: OR, odds ratio; CI, confidence interval; ADL, activities of daily living; CESD-10, 10-item Center for Epidemiologic Studies Depression Scale; mmHg, millimetres of mercury; s, seconds; kg, kilograms; cm, centimetres. Adjusted screening models were adjusted for age, sex, current smoking, and BMI, with each predictor entered separately. *p* values of significant predictors are presented in bold (*p* < 0.05).

**Table 3:** Final complete-case multivariable logistic regression model for subsequent falls.

Predictor	OR (95% CI)	*p* Value
Frailty index, per 0.1-unit increase	1.26 (1.07–1.48)	**0.004**
ADL limitation score, per 1-point increase	1.30 (1.16–1.45)	**<0.001**
Full-tandem balance time, per 10 s increase	0.89 (0.82–0.96)	**0.005**
Age, per 1-year increase	0.98 (0.95–1.00)	0.077
Women vs. men	1.62 (1.17–2.25)	**0.004**
BMI, per 1 kg/m^2^ increase	1.00 (0.97–1.04)	0.891
Current smoking vs. no current smoking	0.75 (0.51–1.09)	0.129
Alcohol use vs. no alcohol use	1.29 (0.96–1.74)	0.096
Upper secondary/vocational vs. less than lower secondary	0.68 (0.43–1.08)	0.101
Tertiary vs. less than lower secondary	0.62 (0.14–2.86)	0.542
Widowed vs. coupled	1.12 (0.82–1.53)	0.464
Other uncoupled vs. coupled	1.52 (0.65–3.53)	0.330
Medication burden, per 1-unit increase	0.96 (0.86–1.07)	0.483

Note. Values are odds ratios with 95% confidence intervals. ADL, activities of daily living; BMI, body mass index; CI, confidence interval; OR, odds ratio. The bold formatting indicates that the predictor was statistically significant.

**Table 4:** Sensitivity analyses examining overlap between frailty and ADL limitation.

Predictor	Model Excluding ADL Limitation OR (95% CI)	*p* Value	Model Excluding Frailty Index OR (95% CI)	*p* Value
Frailty index, per 0.1-unit increase	1.46 (1.27–1.69)	**<0.001**	—	—
ADL limitation score, per 1-point increase	—	—	1.39 (1.25–1.54)	**<0.001**
Full-tandem balance time, per 10 s increase	0.88 (0.81–0.96)	**0.003**	0.89 (0.82–0.96)	**0.005**
Age, per 1-year increase	0.98 (0.95–1.01)	0.121	0.98 (0.95–1.00)	0.092
Women vs. men	1.60 (1.16–2.21)	**0.004**	1.70 (1.23–2.35)	**<0.001**
BMI, per 1 kg/m^2^ increase	1.00 (0.96–1.03)	0.923	1.00 (0.97–1.04)	0.891
Current smoking vs. no smoking	0.74 (0.51–1.07)	0.112	0.76 (0.52–1.10)	0.146
Alcohol use vs. no alcohol use	1.28 (0.95–1.72)	0.102	1.28 (0.95–1.72)	0.108
Upper secondary/vocational vs. less than lower secondary	0.66 (0.42–1.04)	0.071	0.67 (0.43–1.05)	0.084
Tertiary vs. less than lower secondary	0.54 (0.12–2.46)	0.421	0.64 (0.14–2.95)	0.565
Widowed vs. coupled	1.13 (0.83–1.53)	0.444	1.13 (0.83–1.54)	0.429
Other uncoupled vs. coupled	1.60 (0.70–3.65)	0.267	1.53 (0.66–3.53)	0.324
Medication burden, per 1-unit increase	0.93 (0.84–1.03)	0.171	1.07 (0.99–1.16)	0.073

Note. Values are odds ratios with 95% confidence intervals. Both models were adjusted for the remaining final-model covariates. ADL, activities of daily living; BMI, body mass index; CI, confidence interval; OR, odds ratio. The bold formatting indicates that the predictor was statistically significant.

**Table 5:** Multiple-imputation sensitivity analysis for the final multivariable logistic regression model.

Predictor	OR (95% CI)	*p* Value
Frailty index, per 0.1-unit increase	1.27 (1.11–1.45)	**<0.001**
ADL limitation score, per 1-point increase	1.27 (1.16–1.39)	**<0.001**
Full-tandem balance time, per 10 s increase	0.90 (0.83–0.98)	**0.016**
Age, per 1-year increase	0.98 (0.96–1.00)	0.077
Women vs. men	1.37 (1.04–1.81)	**0.023**
BMI, per 1 kg/m^2^ increase	0.99 (0.96–1.03)	0.728
Current smoking vs. no current smoking	0.74 (0.54–1.02)	0.068
Alcohol use vs. no alcohol use	1.17 (0.91–1.52)	0.221
Upper secondary/vocational vs. less than lower secondary	0.65 (0.32–1.30)	0.224
Tertiary vs less than lower secondary	1.33 (0.54–3.31)	0.534
Widowed vs. coupled	1.14 (0.88–1.49)	0.320
Other uncoupled vs. coupled	1.40 (0.69–2.85)	0.346
Medication burden, per 1-unit increase	0.94 (0.85–1.03)	0.174

Note. Values are odds ratios with 95% confidence intervals. The bold formatting indicates that the predictor was statistically significant.

**Table 6:** Sensitivity analysis using a modified frailty index with the ADL component removed.

Predictor	OR (95% CI)	*p* Value
Modified frailty index excluding ADL, per 0.1-unit increase	1.11 (1.00–1.24)	0.056
ADL limitation score, per 1-point increase	1.36 (1.23–1.51)	**<0.001**
Full-tandem balance time, per 10 s increase	0.89 (0.82–0.97)	**0.008**
Age, per 1-year increase	0.98 (0.95–1.00)	0.095
Women vs. men	1.69 (1.22–2.34)	**0.002**
Cleaned BMI, per 1 kg/m^2^ increase	1.00 (0.96–1.04)	0.972
Current smoking vs. no current smoking	0.76 (0.52–1.11)	0.159
Alcohol use vs. no alcohol use	1.28 (0.95–1.73)	0.099
Upper secondary/vocational vs. less than lower secondary	0.48 (0.16–1.16)	0.138
Tertiary vs less than lower secondary	1.13 (0.31–3.31)	0.836
Widowed vs. coupled	1.12 (0.82–1.53)	0.455
Other uncoupled vs. coupled	1.54 (0.63–3.46)	0.318
Medication burden, per 1-unit increase	1.04 (0.95–1.13)	0.418

Note. Values are odds ratios with 95% confidence intervals. The model included 1451 participants. The modified frailty index was recalculated after removing the ADL component from the frailty-index numerator and denominator. The model included the same covariates as the final complete-case model, with ADL limitation retained as a separate predictor. ADL, activities of daily living; BMI, body mass index; CI, confidence interval; OR, odds ratio. The bold formatting indicates that the predictor was statistically significant.

**Table 7:** Sensitivity analysis adding CESD-10 score to the final multivariable model.

Predictor	OR (95% CI)	*p* Value
Frailty index, per 0.1-unit increase	1.25 (1.04–1.50)	**0.018**
ADL limitation score, per 1-point increase	1.32 (1.17–1.49)	**<0.001**
Full-tandem balance time, per 10 s increase	0.86 (0.79–0.94)	**0.001**
Age, per 1-year increase	0.98 (0.95–1.01)	0.230
Women vs. men	1.54 (1.07–2.23)	**0.020**
Cleaned BMI, per 1 kg/m^2^ increase	1.00 (0.96–1.04)	0.875
Current smoking vs. no current smoking	0.82 (0.54–1.23)	0.337
Alcohol use vs. no alcohol use	1.37 (0.98–1.90)	0.065
Upper secondary/vocational vs. less than lower secondary	0.12 (0.01–0.58)	**0.039**
Tertiary vs. less than lower secondary	0.98 (0.21–3.35)	0.981
Widowed vs. coupled	1.17 (0.83–1.65)	0.365
Other uncoupled vs. coupled	1.62 (0.59–4.00)	0.314
Medication burden, per 1-unit increase	0.98 (0.86–1.10)	0.706
CESD-10 score, per 1-point increase	1.02 (1.00–1.04)	0.080

Note. Values are odds ratios with 95% confidence intervals. The model included 1202 participants because CESD-10 had additional missing data. CESD-10 was added to the final complete-case multivariable model as an additional covariate. ADL, activities of daily living; BMI, body mass index; CESD-10, 10-item Center for Epidemiologic Studies Depression Scale; CI, confidence interval; OR, odds ratio. The bold formatting indicates that the predictor was statistically significant.

## Data Availability

The datasets analysed for the present study are available in the CHARLS repository at http://charls.pku.edu.cn.

## Abbreviations

The following abbreviations are used in this manuscript:

ADL	Activities of daily living
AUC	Area under the receiver operating characteristic curve
BMI	Body mass index
BP	Blood pressure
CESD-10	10-item Center for Epidemiologic Studies Depression Scale
CHARLS	China Health and Retirement Longitudinal Study
CI	Confidence interval
Df	Degrees of freedom
FI	Frailty index
FTB	Full-tandem balance time
GVIF	Generalized variance inflation factor
IADL	Instrumental activities of daily living
IQR	Interquartile range
IRB	Institutional Review Board
MB	Medication burden
OR	Odds ratio
ROC	Receiver operating characteristic
SD	Standard deviation
SPSS	Statistical Package for the Social Sciences
STROBE	Strengthening the Reporting of Observational Studies in Epidemiology
VIF	Variance inflation factor

## Appendix A. Supplementary Analyses

This appendix contains supplemental analyses supporting the main manuscript.

**Table A1:** Deficits included in frailty index.

Domain	Deficit Item	Coding Used in Frailty Index
Chronic disease	High blood pressure	0 = no; 1 = yes
Chronic disease	Diabetes	0 = no; 1 = yes
Chronic disease	Dyslipidemia	0 = no; 1 = yes
Chronic disease	Heart problem	0 = no; 1 = yes
Chronic disease	Stroke	0 = no; 1 = yes
Chronic disease	Arthritis	0 = no; 1 = yes
Chronic disease	Lung disease	0 = no; 1 = yes
Chronic disease	Asthma	0 = no; 1 = yes
Chronic disease	Cancer	0 = no; 1 = yes
Chronic disease	Kidney disease	0 = no; 1 = yes
Chronic disease	Liver disease	0 = no; 1 = yes
Chronic disease	Stomach or digestive disease	0 = no; 1 = yes
Chronic disease	Psychiatric or emotional problem	0 = no; 1 = yes
Chronic disease	Memory-related disease	0 = no; 1 = yes
Mobility limitation	Mobility limitation item 1	0 = no limitation; 1 = limitation
Mobility limitation	Mobility limitation item 2	0 = no limitation; 1 = limitation
Mobility limitation	Mobility limitation item 3	0 = no limitation; 1 = limitation
Functional limitation	ADL limitation	6-item ADL score rescaled to 0–1
Functional limitation	IADL limitation	5-item IADL score rescaled to 0–1
Depressive symptoms	CESD-10 depressive symptoms	CESD-10 score rescaled to 0–1
Sleep-related symptoms	Sleep disturbance	0 = no deficit; 1 = deficit
Cognition	Orientation	Orientation deficit rescaled to 0–1
Cognition	Figure drawing	0 = correct/no deficit; 1 = incorrect/deficit
Physical activity	Low physical activity	0 = physically active; 1 = low/no physical activity

**Table A2:** Comparison of participants included in and excluded from the complete-case regression model.

Characteristic	Included (n = 1451)	Excluded (n = 481)	*p* Value
**Follow-up falls, n (%)**			0.394
No falls	1098 (75.7)	354 (73.6)	
Falls	353 (24.3)	127 (26.4)	
Age, years, mean (SD)	71.81 (5.84)	72.92 (6.94)	**0.001**
**Sex, n (%)**			**0.020**
Men	730 (50.3)	212 (44.1)	
Women	721 (49.7)	269 (55.9)	
Frailty index, mean (SD)	0.257 (0.124)	0.277 (0.133)	**0.004**
ADL limitation score, mean (SD)	0.55 (1.15)	0.92 (1.58)	**<0.001**
IADL limitation score, mean (SD)	0.68 (1.17)	1.09 (1.52)	**<0.001**
Medication burden, mean (SD)	1.57 (1.64)	1.70 (1.72)	0.143
BMI, kg/m^2^, mean (SD)	22.80 (3.60)	23.29 (4.68)	0.203
Full-tandem balance time, mean (SD)	4.22 (1.82)	4.77 (1.59)	0.343
**Current smoking, n (%)**			0.103
No current smoking	1068 (73.6)	367 (76.3)	
Current smoking	383 (26.4)	113 (23.5)	
**Alcohol use in the last year, n (%)**			0.057
No alcohol use	997 (68.7)	349 (72.6)	
Alcohol use	454 (31.3)	131 (27.2)	
**Education level, n (%)**			0.138
Less than lower secondary	1265 (87.2)	402 (83.6)	
Upper secondary/vocational	172 (11.9)	73 (15.2)	
Tertiary	14 (1.0)	6 (1.2)	
**Marital status, n (%)**			**0.369**
Coupled	1054 (72.6)	334 (69.4)	
Widowed	366 (25.2)	134 (27.9)	
Other uncoupled	31 (2.1)	13 (2.7)	

Values are presented as mean (SD) for continuous variables and n (%) for categorical variables. *p* values compare participants included in the complete-case model with those excluded because of missing covariate data. The bold formatting indicates that the predictor was statistically significant.

**Table A3:** Collinearity diagnostics for the final complete-case model.

Predictor	GVIF	Df	Adjusted GVIF
Frailty index, per 0.1-unit increase	2.508	1	1.584
ADL limitation score	1.291	1	1.136
Full-tandem balance time, per 10 s increase	1.488	1	1.220
Age	1.618	1	1.272
Sex	1.637	1	1.279
Cleaned BMI	1.113	1	1.055
Current smoking	1.426	1	1.194
Alcohol use in the last year	1.173	1	1.083
Education level	1.068	2	1.017
Marital status	1.226	2	1.052
Medication burden	2.178	1	1.476

Note. GVIF, generalized variance inflation factor; Df, degrees of freedom; ADL, activities of daily living; BMI, body mass index. Adjusted GVIF was calculated as GVIF^(1/[2 × Df]) for multi-level categorical predictors.

**Table A4:** Correlation matrix of selected continuous clinical predictors.

Variable	FI	ADL	IADL	MB	FTB	BMI	Age
FI	1.000	0.444 *	0.344 *	0.677 *	−0.088 *	0.121 *	0.041
ADL	0.444 *	1.000	0.439 *	0.228 *	−0.102 *	0.014 *	0.098 *
IADL	0.344 *	0.439 *	1.000	0.157 *	−0.202 *	−0.048 *	0.173 *
MB	0.677 *	0.228 *	0.157 *	1.000	−0.038	0.215	−0.031
FTB	−0.088 *	−0.102 *	−0.202 *	−0.038	1.000	0.058 *	−0.607 *
BMI	0.121 *	0.014	−0.048	0.215 *	0.058 *	1.000	−0.124 *
Age	0.041	0.098 *	0.173 *	−0.031	−0.607 *	−0.124 *	1.000

Note. Values are Spearman correlation coefficients. *, *p* < 0.05. FI, frailty index; ADL, activities of daily living; IADL, instrumental activities of daily living; MB, medication burden; FTB, full-tandem balance time; BMI, body mass index. The full-tandem balance time and BMI variables correspond to the cleaned/scaled variables used in the complete-case analysis.

**Table A5:** Sex-specific univariable and adjusted screening logistic regression analyses of baseline predictors of falls among men.

Predictor	Comparison/Unit	Unadjusted Models		Adjusted Models	
		OR (95% CI)	*p* Value	OR (95% CI)	*p* Value
Frailty index	Per 0.1-unit increase	1.32 (1.13–1.55)	<0.001	1.32 (1.13–1.55)	<0.001
ADL limitation score	Per 1-point increase	1.45 (1.24–1.68)	<0.001	1.45 (1.24–1.69)	<0.001
IADL limitation score	Per 1-point increase	1.40 (1.19–1.65)	<0.001	1.41 (1.19–1.66)	<0.001
Full-tandem balance time	Per 10 s increase	0.92 (0.82–1.03)	0.127	0.88 (0.76–1.03)	0.105
Age	Per 1-year increase	1.01 (0.98–1.04)	0.615	1.01 (0.98–1.04)	0.658
Cleaned BMI	Per 1 kg/m^2^ increase	0.99 (0.93–1.05)	0.689	0.99 (0.93–1.05)	0.699
Current smoking	Current smoking vs. no current smoking	0.91 (0.60–1.38)	0.652	0.90 (0.59–1.38)	0.644
Alcohol use in the last year	Yes vs. no	1.18 (0.77–1.80)	0.442	1.16 (0.76–1.79)	0.490
Education level	Upper secondary/vocational training vs. less than lower secondary	0.74 (0.28–1.93)	0.534	0.73 (0.28–1.94)	0.531
Education level	Tertiary education vs. less than lower secondary	0.86 (0.19–3.92)	0.842	0.82 (0.18–3.80)	0.803
Marital status	Widowed vs. married/partnered	0.83 (0.36–1.91)	0.667	0.77 (0.33–1.81)	0.555
Marital status	Other uncoupled vs. married/partnered	2.19 (0.88–5.47)	0.094	2.25 (0.89–5.70)	0.088
Medication burden	Per medication	1.06 (0.94–1.19)	0.328	1.07 (0.95–1.20)	0.279

**Table A6:** Sex-specific univariable and adjusted screening logistic regression analyses of baseline predictors of falls among women.

Predictor	Comparison/Unit	Unadjusted Models		Adjusted Models	
		OR (95% CI)	*p* Value	OR (95% CI)	*p* Value
Frailty index	Per 0.1-unit increase	1.41 (1.24–1.60)	<0.001	1.42 (1.24–1.62)	<0.001
ADL limitation score	Per 1-point increase	1.42 (1.25–1.61)	<0.001	1.43 (1.26–1.62)	<0.001
IADL limitation score	Per 1-point increase	1.19 (1.06–1.34)	0.003	1.20 (1.07–1.35)	0.003
Full-tandem balance time	Per 10 s increase	0.89 (0.82–0.96)	0.005	0.86 (0.79–0.95)	0.002
Age	Per 1-year increase	1.01 (0.98–1.03)	0.720	1.01 (0.98–1.03)	0.669
Cleaned BMI	Per 1 kg/m^2^ increase	1.01 (0.97–1.06)	0.526	1.01 (0.97–1.06)	0.541
Current smoking	Current smoking vs. no current smoking	0.62 (0.20–1.92)	0.412	0.65 (0.21–2.00)	0.450
Alcohol use in the last year	Yes vs. no	1.11 (0.70–1.76)	0.656	1.15 (0.72–1.84)	0.549
Education level	Upper secondary/vocational training vs. less than lower secondary	0.00 (0.00–Inf)	0.977	0.00 (0.00–Inf)	0.977
Education level	Tertiary education vs. less than lower secondary	1.43 (0.24–8.62)	0.696	1.34 (0.22–8.15)	0.748
Marital status	Widowed vs. married/partnered	1.42 (0.96–2.10)	0.081	1.45 (0.96–2.18)	0.078
Marital status	Other uncoupled vs. married/partnered	0.18 (0.02–1.40)	0.102	0.18 (0.02–1.37)	0.097
Medication burden	Per medication	1.19 (1.08–1.30)	<0.001	1.19 (1.09–1.31)	<0.001

**Table A7:** Sex-specific final multivariable logistic regression models of baseline predictors of falls.

Predictor	Comparison/Unit	Men		Women	
		OR (95% CI)	*p* Value	OR (95% CI)	*p* Value
Frailty index	Per 0.1-unit increase	1.35 (1.06–1.73)	0.017	1.23 (0.99–1.52)	0.063
ADL limitation score	Per 1-point increase	1.30 (1.09–1.56)	0.004	1.29 (1.12–1.50)	<0.001
Full-tandem balance time	Per 10 s increase	0.87 (0.74–1.01)	0.076	0.89 (0.81–0.99)	0.026
Age	Per 1-year increase	0.97 (0.93–1.02)	0.195	0.97 (0.94–1.01)	0.107
Current smoking	Current smoking vs. no current smoking	0.89 (0.57–1.39)	0.611	0.51 (0.16–1.67)	0.268
Cleaned BMI	Per 1 kg/m^2^ increase	1.00 (0.94–1.06)	0.975	1.00 (0.96–1.05)	0.879
Alcohol use in the last year	Yes vs. no	1.19 (0.76–1.86)	0.435	1.23 (0.75–2.02)	0.416
Education level	Upper secondary/vocational training vs. less than lower secondary	0.68 (0.25–1.86)	0.452	0.00 (0.00–Inf)	0.976
Education level	Tertiary education vs. less than lower secondary	0.75 (0.16–3.59)	0.719	1.99 (0.32–12.42)	0.462
Marital status	Widowed vs. married/partnered	0.88 (0.37–2.08)	0.765	1.55 (1.01–2.38)	0.043
Marital status	Other uncoupled vs. married/partnered	1.93 (0.72–5.19)	0.191	0.22 (0.03–1.78)	0.157
Medication burden	Per medication	0.86 (0.72–1.02)	0.086	1.02 (0.88–1.18)	0.798
